# Genome resources for three modern cotton lines guide future breeding efforts

**DOI:** 10.1038/s41477-024-01713-z

**Published:** 2024-05-30

**Authors:** Avinash Sreedasyam, John T. Lovell, Sujan Mamidi, Sameer Khanal, Jerry W. Jenkins, Christopher Plott, Kempton B. Bryan, Zhigang Li, Shengqiang Shu, Joseph Carlson, David Goodstein, Luis De Santiago, Ryan C. Kirkbride, Sebastian Calleja, Todd Campbell, Jenny C. Koebernick, Jane K. Dever, Jodi A. Scheffler, Duke Pauli, Johnie N. Jenkins, Jack C. McCarty, Melissa Williams, LoriBeth Boston, Jenell Webber, Joshua A. Udall, Z. Jeffrey Chen, Fred Bourland, Warwick N. Stiller, Christopher A. Saski, Jane Grimwood, Peng W. Chee, Don C. Jones, Jeremy Schmutz

**Affiliations:** 1 Genome Sequencing Center, HudsonAlpha Institute for Biotechnology, Huntsville, AL USA; 2 DOE Joint Genome Institute, Berkeley, CA USA; 3 Department of Crop and Soil Sciences and Institute of Plant Breeding, Genetics, and Genomics, University of Georgia, Tifton, GA USA; 4 Department of Plant and Environmental Sciences, Clemson University, Clemson, SC USA; 5 Department of Molecular Biosciences, The University of Texas at Austin, Austin, TX USA; 6 School of Plant Sciences, University of Arizona, Tucson, AZ USA; 7 USDA-ARS, Coastal Plains Soil Water and Plant Research Center, Florence, SC USA; 8 Department of Crop, Soil and Environmental Sciences, Auburn University, Auburn, AL USA; 9 Texas A&M AgriLife Research, Lubbock, TX USA; 10 USDA-ARS, Crop Genetics Research Unit, Stoneville, MS USA; 11 USDA-ARS, Genetics and Sustainable Agriculture Research Unit, Mississippi State, MS USA; 12 USDA-ARS, Crop Germplasm Research Unit, College Station, TX USA; 13 Northeast Research and Extension Center (NEREC), University of Arkansas, Keiser, AR USA; 14 CSIRO Agriculture and Food Cotton Research Unit, Narrabri, New South Wales Australia; 15 Agriculture and Environmental Research Cotton Incorporated, Cary, NC USA; 16 Present Address: Pee Dee Research and Education Center, Clemson University, Florence, SC USA

**Keywords:** Plant breeding, Plant genetics

## Abstract

Cotton (*Gossypium hirsutum* L.) is the key renewable fibre crop worldwide, yet its yield and fibre quality show high variability due to genotype-specific traits and complex interactions among cultivars, management practices and environmental factors. Modern breeding practices may limit future yield gains due to a narrow founding gene pool. Precision breeding and biotechnological approaches offer potential solutions, contingent on accurate cultivar-specific data. Here we address this need by generating high-quality reference genomes for three modern cotton cultivars (‘UGA230’, ‘UA48’ and ‘CSX8308’) and updating the ‘TM-1’ cotton genetic standard reference. Despite hypothesized genetic uniformity, considerable sequence and structural variation was observed among the four genomes, which overlap with ancient and ongoing genomic introgressions from ‘Pima’ cotton, gene regulatory mechanisms and phenotypic trait divergence. Differentially expressed genes across fibre development correlate with fibre production, potentially contributing to the distinctive fibre quality traits observed in modern cotton cultivars. These genomes and comparative analyses provide a valuable foundation for future genetic endeavours to enhance global cotton yield and sustainability.

## Abstract (web summary)

Analyses of three newly sequenced modern cultivar cotton genomes revealed sequence and structural variation alongside traces of ancient and ongoing introgressions. Moreover, transcriptome analysis pointed at unique fibre quality traits of cultivars.

## Main

Domesticated around 8,000 years ago^1^, cotton cultivation began with a reduction in genetic diversity during the initial selection process, but cultivated germplasm has since diversified from this limited gene pool. Genetic diversity has been further constrained by recent strong selection within modern breeding programmes, which have produced cultivars that represent the bulk of current global cotton production. This recent and strong selection has further subdivided cotton genetic diversity: modern germplasm is distinct from unimproved cultivars and other sources of molecular variation. Therefore, cotton breeding efforts would particularly benefit from enhanced genome-enabled breeding and biotechnology.

Novel climates, pathogens and other environmental stressors are decreasing yield stability and impeding improvement efforts across many crops. Recently, breeders have successfully met these challenges using molecular and genome-enabled tools to improve existing cultivars and develop new modern varieties. Such efforts have been particularly powerful in species with mature genomic resources, such as rice, tomato, maize and wheat^2–8^. In some cases, rigorous multi-year breeding efforts have been integrated with genomic tools and datasets to quickly develop well-adapted cultivars to new environments. For example, rice breeders have integrated molecular variation within the submergence-tolerant 1 locus (*Sub1A*) with traditional efforts to accelerate the release of locally adapted flood-tolerant cultivars^9^. However, mimicking this success story is not possible in many other plant breeding programmes, in part because of limited genetic diversity and a lack of high-confidence sequence information for high-value molecular targets such as *Sub1A*. Cotton is such a system, where high levels of sequence divergence between hybridizing species and a reference genome that is highly diverged from elite germplasm have impeded biotechnology-driven precision breeding efforts.

At present, cotton improvement efforts rely largely on traditional breeding approaches, which have led to improved fibre yield and quality^10–14^, among other desirable traits. However, achieving additional genetic gains through traditional breeding methods may prove challenging: genetic uniformity among modern cultivars simultaneously limits the efficacy of selection and escalates the impacts of disease and climatic stress. For example, early molecular breeding strategies have shown that genomic selection can improve efficiency^15^. However, a deeper understanding of the genetic make-up of parental lines is required for appropriate selection of progeny in the early stages of the breeding cycle.

Cotton biotechnology is further complicated by the use of the ‘TM-1’ historical genetic standard for ongoing molecular enquiries. TM-1 has served the cotton community well as the reference genotype since 1970^16^ but is no longer used in any breeding programmes because of its inferior yield and fibre quality traits compared with modern germplasm and cultivars^17,18^. Furthermore, the current but outdated TM-1 reference genome, which was most recently updated in 2018^19^, is not well suited to the repetitive and polyploid cotton genome.

To facilitate modern molecular breeding and build a strong foundation for accelerated cotton improvement, we generated chromosome-scale reference genomes for three public modern cotton cultivars: ‘UGA230’, ‘UA48’ and ‘CSX8308’ (see Methods for detailed descriptions of these cultivars). UA48 is adapted to higher-latitude US fields with strong blight resistance and exceptional fibre quality. UGA230 is broadly adapted to southern North American conditions with high yield in long growing seasons and some of the longest fibres of any cultivar. CSX8308 is an okra-leaf cultivar adapted to Australian conditions with strong resistance to fusarium wilt. In addition to these three cultivars, we updated the reference genome assembly and annotation for TM-1. Genome-wide comparison of reference assemblies revealed sequence, structural and gene content variation among the four genotypes, including introgression of highly diverged sequence from the related ‘Pima’ *Gossypium barbadense* cotton species. Combined with the identification of introgressed regions, structural variation and transcriptional response, our analyses and genome resources provide a foundation for the cotton research community that should facilitate and accelerate future precision breeding efforts.

## Results

### A more complete reference genome for cultivated cotton

**Fig. 1:**
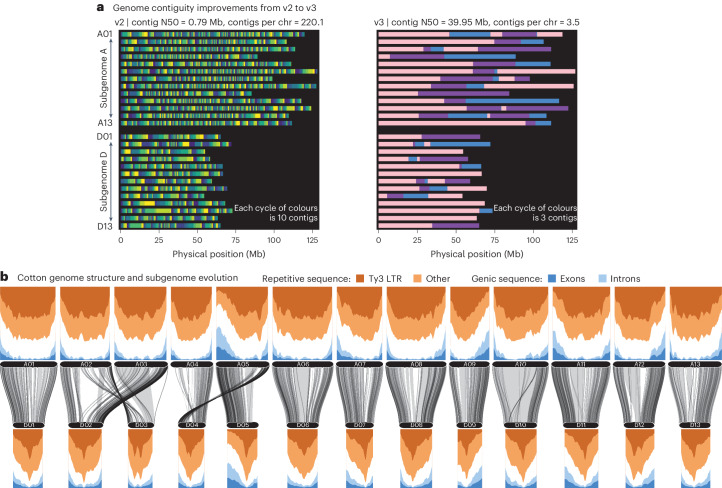
Structure and contiguity of the TM-1 cotton reference genome. The v2 and v3 reference genome sequences were subjected to contig position mapping by GENESPACE. **a**, The contigs in each genome (v2, left; v3, right) as a continuous block of a single colour. Given the substantial differences in contiguity, a continuous yellow–blue palette with ten colours was selected for v2, while a discrete three-colour sequence (pink, purple, blue) was used for v3. **b**, The difference in genome architecture between the A (top) and D (bottom) subgenomes of the tetraploid TM-1 v3 cotton. Repeat and gene density were hierarchically inferred, classifying the genomes into exons, Ty3 repeats, other repeats (from RepeatMasker), introns and other (white). Sliding windows (5 Mb width, 1 Mb steps) are plotted. Decomposed blocks of alignments from minimap2 are shown between the two subgenomes.

The cotton breeding and genetics community currently relies on the v2 reference sequence of TM-1 as the foundation for sequence and marker discovery. While serviceable, the TM-1 v2 reference sequence suffers two major limitations. First, the previous assembly was unable to accurately distinguish sequences in the substantial and highly repetitive pericentromeres of the cotton genome, which produced a fragmented assembly with 5,723 contigs (Fig. 1a). To provide a foundation for further cotton comparative genomics and reference-based approaches, we reconstructed the TM-1 reference genome using deep (116.7×) PacBio CLR, 55.0× Illumina sequence polishing (Methods) and Hi-C scaffolding (172×). Heterozygosity tends to be very low in inbred tetraploid cotton cultivars, and TM-1 is no exception with 12,173 heterozygous sites (single-nucleotide polymorphisms (SNPs) or insertions and deletions (indels) across the 2,154 million callable bases (5.6 heterozygous sites per megabase). This heterozygosity also justifies a haploid genome assembly representation and the use of continuous long read (CLR) sequencing technology.

The resulting v3 TM-1 reference genome represents 26 chromosomes with only 91 contigs (mean of 2.1 gaps per chromosome, contig N50 of 40.0 million bases, ‘megabases’, ‘Mb’), a 63-fold improvement in contiguity compared with v2 (5,703 total gaps in the v2 chromosomes). This level of contiguity improvement also applies to the more recently updated Huang et al. genome assembly, which consists of 1,235 contigs and a contig N50 of 5.02 Mb^20^. The improved contiguity combined with Hi-C contact maps revealed 35 within-chromosome inversions (totalling 122 Mb) between the v2 and v3 assemblies, probably due to miss-assemblies in the v2 release. To facilitate information transfer, we constructed a synteny map between the two genome versions (Supplementary Data 1). The corrected inversions, increased per-base sequencing depth, improved accuracy and substantial reduction in gapped sequence in the v3 TM-1 genome result in a superior reference genome that will better support breeding and biotechnology goals.

The high level of contiguity of the TM-1 v3 genome in previously fragmented repetitive regions permitted much higher confidence tests of the structure of cotton genomes. Overall, the TM-1 genome is very repetitive: 1,603 Mb (70.8%) of the 2,265 Mb genome sequences are repetitive, while 246 Mb (10.9%) are in protein-coding transcripts, and an astounding 776.5 Mb (34.3%) of the genome is made purely of Ty3 repeats. However, this repeat content is not uniformly distributed: repeat and gene density varies considerably within and among chromosomes. Most of the genes reside on chromosome arms, while pericentromeres are rife with repeat elements (Fig. 1b).

**Extended Data Fig. 1:**
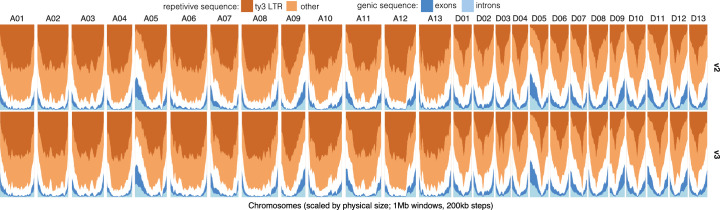
Repeat and gene content comparison between TM-1 reference genome versions, v2 and v3. The difference in genome architecture between the v2 (top) and v3 (bottom) versions. Repeat and gene density were inferred hierarchically, classifying the genomes as in exons, ty3 repeats, other repeats (from repeatMasker), introns, other (white). Sliding windows (5 Mb width, 1 Mb steps) are plotted. Source data

The two cotton subgenomes (‘A’ and ‘D’) show highly diverged patterns of gene and repeat density: the larger A (1,429.26 Mb) and more compact D (835.92 Mb) subgenomes contain very similar gene content (121.8 Mb and 124.8 Mb, respectively; Fig. 1b). The nearly twofold difference in subgenome size is instead primarily driven by repeat content evolution where the A subgenome has 2.1× more repeats overall (1,076.2 Mb versus 501.6 Mb) and nearly three times as many Ty3 repeats (577.0 Mb versus 196.2 Mb), but nearly identical Ty1 repeat content (48.7 Mb versus 47.7 Mb). While these observations largely mirror those of other groups^20^ and using the previous reference genome (Extended Data Fig. 1), the substantial improvement in contiguity across repetitive regions demonstrates that the observed patterns of subgenome variation are not sequencing artefacts. The more complete v3 sequences of the TM-1 genotype will provide a more accurate foundation for genotyping because the full complement of repetitive sequences is known and can be properly controlled for.

### Cotton germplasm necessitates modern cultivar references

**Fig. 2:**
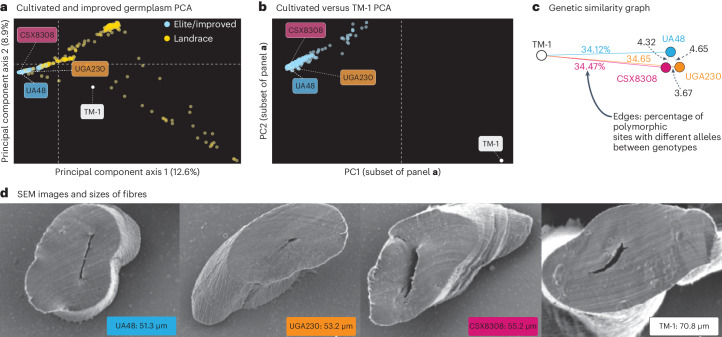
Molecular and phenotypic variation between TM-1 and the three modern cultivars. **a**,**b**, Principal components were calculated through principal component analysis (PCA) from 7.3 million SNPs across 218 landrace, 228 improved/modern and TM-1 genotypes in the full set of materials (**a**) and the TM-1 and improved/modern lines (**b**). **c**, The same set of polymorphic SNPs was used to calculate genetic distances among the polishing libraries of the four reference genomes; the graph of these distances with *x*–*y* positions derived from the distances (multidimensional scaling (MDS) coordinates). **d**, Scanning electron microscopy images and data from representative fibres that had a circumference close to the mean of each genotype (*n* = 60). The numerical value within each image refers to the exact circumference of the fibre.

TM-1 was originally chosen as the cotton reference because of its importance in genetic and cytogenetic research^21^. TM-1 also fortuitously occupies a relatively equidistant position relative to a set of 400 genotypes selected to represent most of genetic diversity in cotton (Fig. 2a), making it an ideal reference for short-read mapping across different cotton varieties. However, current breeding programmes view TM-1 as an obsolete genotype offering limited improvement value. Consistent with this observation, genomic sequences (Fig. 2b,c) and fibre traits (Fig. 2d) of improved and modern cultivars have markedly diverged from the TM-1 lineage. As cotton has a large, duplicated and highly repetitive genome, the phenotypic and sequence differences between modern genotypes and TM-1 are sufficiently large enough to make it problematic to determine trait-associated targets for crop improvement.

**Extended Data Fig. 2:**
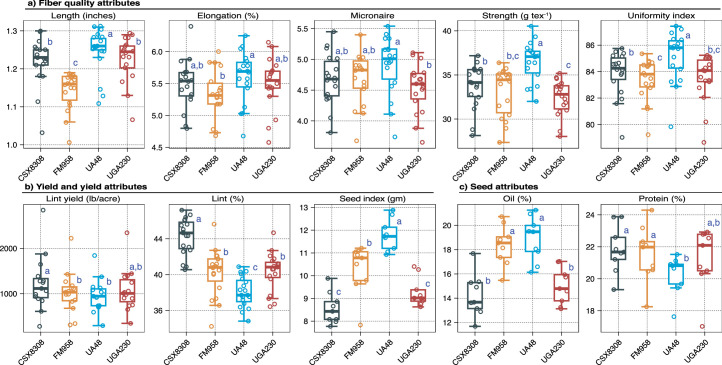
Biometric parameters of cotton fibre. **a**, Quality, **b**, yield and **c**, seed attributes of four cotton cultivars including CSX8308, FM958 (a commercial variety), UA48, and UGA230. The box plots indicate the median (the line within the box); the lower and upper edges of the boxes correspond to the 25th and 75th percentiles of each groups’ distribution of values with whiskers extending to ±1.5× interquartile range (IQR); circles represents a least squares (LS) trait mean for each cultivar (*n* = 4, biologically independent samples) in an environment; alphabets represent significance of contrasts using Tukey’s HSD test - common alphabets within charts are not different at *P* < 0.05. Trait-specific details of number of environments (n) and the range of *P* values (if multiple) at which contrasts between cultivars were declared significant for i) Length (inches): n = 18; *P* < 0.014–0.0001; ii) Elongation (%): n = 18; *P* < 0.008; iii) Micronaire: n = 18; *P* < 0.0002–0.036; iv) Strength (g tex-1): n = 18; *P* < 0.0001–0.0003, v) Uniformity index: n = 18; *P* < 0.0001–0.028, vi) Lint yield (lb/acre): n = 15; *P* < 0.00039–0.009, (vii) Lint (%): n = 19; *P* < 0.0001, viii) Seed index: n = 9; *P* < 0.0001–0.01, ix) Oil (%): n = 9; *P* < 0.0001–0.009, and x) Protein (%): n = 9; *P* < 0.003–0. 019, respectively. Source data

Beginning in 2018, collaborators across US and Australian breeding programmes selected three distinct cultivars as central targets for reference genomes: (1) UGA230, a southeastern conventional upland cotton cultivar adapted to US conditions, (2) UA48, an early-maturing and disease-resistant cultivar and (3) CSX8308, an okra-leaf high-yielding cultivar with broad adaptation across Australian cotton-growing regions. Importantly, these three genomes cover many important breeding gene pools: UGA230 has fine fibres, high yield potential and adaptation to regions with long growing seasons such as the southeastern US Cotton Belt; UA48 has early maturity and high fibre strength and length; and CSX8308 is adapted to Australian conditions with very high gin turnout and excellent bacterial blight resistance^22^. We validated these traditional classifications by assessing fibre quality and yield traits (Fig. 2d, Extended Data Fig. 2 and Extended Data Table 1) of the three modern cultivars and the experimental reference commercial cultivar ‘FM958’ across nine locations in the USA. While CSX8308 has the highest lint yield and gin turnout (lint per cent), UA48 has higher lint length, lint strength and larger seeds^23^. Alternatively, UGA230 has the lowest micronaire, which is an indirect measure of lint fineness by relating the air permeability of compressed cotton fibres. In this study, the lint yield ranged from 950 lb per acre to 1,225 lb per acre across the four cultivars, contrasting with the lower yield of 737.83 lb per acre (827 kg ha^−^^1^) observed for TM-1^24^. Furthermore, the lint percentage ranged from 37% to 44% in our study, compared with the reported figures of 30.49% (ref. ^24^) and 32.76% (ref. ^18^). Given these suboptimal fibre metrics, TM-1 can be classified as outdated germplasm in contemporary breeding programmes. Scanning electron microscopy (SEM) analysis of individual fibres provided clear evidence that the three modern cultivars have much finer fibres with smaller circumference (mean ± s.e.m.: 53.23 ± 1.12 μM) compared with TM-1 (70.8 μM) (Fig. 2d).

**Fig. 3:**
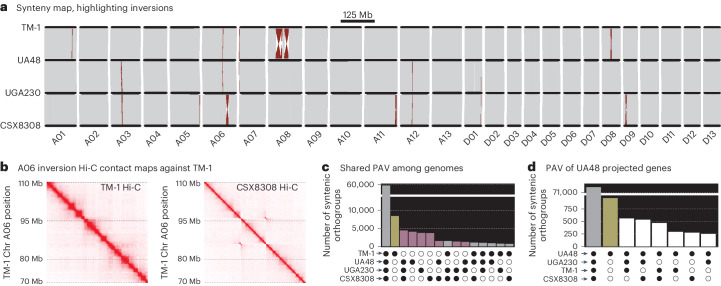
Synteny and PAV across four cotton genomes. **a**, Completely collinear (grey), inverted (red) and PAV (white wedges) sequences are plotted on a common coordinate system across the genomes. **b**, Zoomed-in contact maps of both TM-1 (left) and CSX8308 (right) Hi-C libraries mapped to the TM-1 reference are shown to highlight the chromosome A06 inversion found only in CSX8308. The off-diagonal ‘hourglass’ contacts in CSX8308 clearly confirm the presence of this inversion relative to TM-1. **c**, Gene family PAV within genomes is presented. Gene families private to TM-1 (yellow) and the modern cultivars (pink) are highlighted. **d**, Gene family PAV for ‘liftover’ gene model projection from the UA48 annotation onto the other three genomes demonstrates that hundreds of gene sequences are completely missing across the genomes.

We constructed reference genomes for each of the three lines using identical methods as TM-1 V3, yielding genomes with similar levels of completeness, accuracy and contiguity (Fig. 3a, Table 1 and Extended Data Table 2). Combined, these four assemblies are among the most complete of any plant species with large (2,276–2,294 Mb), polyploid and repetitive genomes. To complement the genome sequences and provide direct support for candidate gene discovery, we built a complete genome annotation for all four genotypes, integrating genotype-specific gene expression and homology support. Overall, we sequenced RNA from 74 libraries for five tissues and a fibre development time course for each genome. Our annotation method produced gene sets with higher completeness (BUSCO (v.5.5)^25^ 98.3–99.0%) than the existing Huang TM-1 reference (97.8%). Combined with a better assembly, it appears that the new annotations capture substantial gene presence–absence variation (PAV) and copy number variation (CNV): 254,581 genes were found in phylogenetically hierarchical orthogroups (produced by OrthoFinder) that spanned all four references, while 47,874 genes were found in orthogroups that were absent in one or more genomes (Fig. 3b). This newly discovered gene PAV and CNV provide new genetic diversity targets for cotton improvement.

**Table 1:** Genome assembly and annotation statistics for three modern cotton cultivars and TM-1

	UGA230	UA48	CSX8308	TM-1 v3	TM-1 v2	TM-1 (Huang)
Assembly size (Mb)	2,265.53	2,253.01	2,269.21	2,265.18	2,305.62	2,290.43
Number of contigs	201	607	207	91	5,723	1,235
Contig N50 (Mb)	27.44	8.14	29.87	39.95	0.78	5.02
Assembly BUSCO (%)	99.5	99.6	99.5	99.5	99.5	99.5
Genome in chromosomes (%)	99.52	98.20	99.62	99.43	98.96	99.16
Number of genes	75,412	75,775	75,605	75,663	74,902	74,350
Alternative transcripts	37,679	36,185	37,450	33,905	31,745	Not available
Annotation BUSCO (%)	99.0	98.3	98.5	98.6	98.5	97.8

### Diverse cultivated cotton genomes permit evolution tests

**Extended Data Fig. 3:**
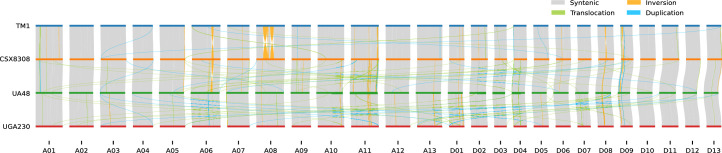
Syntenic regions and large structural variations between TM-1 and modern cotton lines (CSX8308, UA48 and UGA230). Vertical lines connecting chromosomes represent syntenic (gray), inverted (orange), translocated (green) and duplicated (blue) regions.

**Extended Data Fig. 4:**
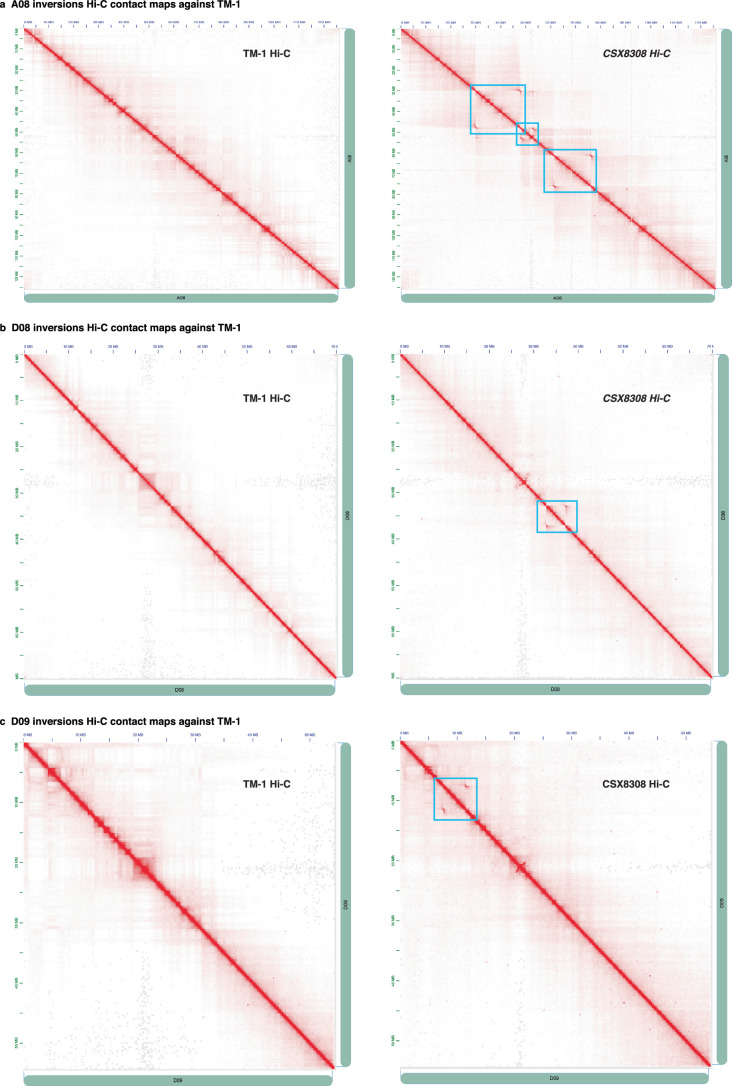
Hi-C contact maps of the TM-1 reference genome. TM-1 (left) and CSX8308 (right) Hi-C libraries mapped to the TM-1 reference are shown for chromosomes **a**, A08, **b**, D08 and **c**, D09. The off-diagonal ‘hourglass’ contacts highlighted in CSX8308 confirm the presence of inversions in that genome relative to TM-1.

**Extended Data Fig. 5:**
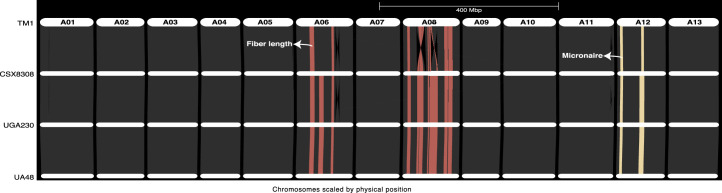
Tracking fibre related QTL regions across cotton cultivars. Fibre length and micronaire QTL regions discovered by Yang et al.^29^ tracked across sequenced cultivars. Fibre length QTL overlaps the large inversions on chromosome A08.

Despite known limited single-nucleotide sequence variation^26,27^, breeders may be able to target other forms of molecular diversity. For example, sequence rearrangements and other structural variations (including inversions and deletions) and gene family CNV and PAV may be important sources of heritable trait variance. We used GENESPACE^28^ to analyse these forms of larger-scale genetic variation, which may have been targets for improvement during selective breeding. Overall, the four cotton genomes were highly collinear (Fig. 3a and Extended Data Fig. 3) with no major translocations and only a mean of 10 large inversions (>40 kb), which contained an average of 31 Mb of sequence between any two pairs of genomes. All major inversions were confirmed through reciprocal Hi-C mapping (Fig. 3b and Extended Data Fig. 4). Indeed, >98.4% of all sequences were fully collinear between each pair of genomes. Despite strong collinearity, large inversions could underlie trait variation in cultivated cotton. For example, fibre length quantitative trait loci (QTL) discovered previously^29^ overlaps the large inversions on chromosome A08 (Extended Data Fig. 5). While our sample size precludes any causal inference that would connect structural variations to traits, the synteny map across our four reference genomes provides a resource for breeders to track and find variants within genomic regions of interest.

**Extended Data Fig. 6:**
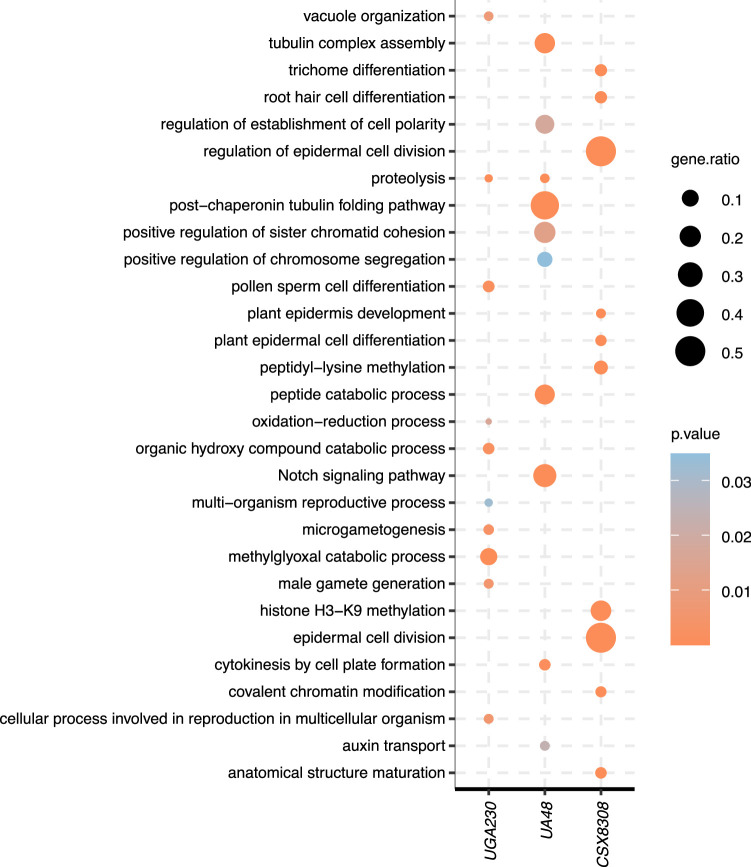
Overrepresented Gene Ontology terms (biological processes). Top 10 significant GO terms (Fisher’s exact test, *P* < 0.05) representing an aggregate of those enriched among modern cotton lines specific expanded gene families. Source data

Given higher sequence confidence in the new references, we sought to conduct a thorough examination of gene content evolution in cotton. First, we explored gene family expansion and contraction by integrating PLAZA^30^ gene family information with orthogroups; 18 (UGA230, 324 genes), 19 (UA48, 783 genes) and 9 (CSX8308, 199 genes) gene families were considerably expanded in each genome. These expanded gene families were enriched in functional annotations related to reproduction, specifically pollen cell differentiation in UGA230, tubulin complex assembly and auxin transport in UA48, and epidermal cell division, trichome differentiation and, strikingly, methylation and chromatin modification in CSX8308, which have been previously shown to influence both fibre cell number and length^31^ (Extended Data Fig. 6).

Across the four genomes, gene PAV-based clustering mirrored SNP-based clustering (Fig. 2b), where the three modern cultivar genomes have more similar gene content to each other than to TM-1 (Fig. 3c). Crucially, we discovered 15,472 syntenic gene families (18.02% of all syntenic orthogroups) that were absent in TM-1 but present in one or more of the modern cultivar genomes. As expected, given its phylogenetically diverged position, TM-1 showed the largest number of private gene sets (6,684, the modern cultivars ranged from 2,825 to 4,674; Fig. 3c). Conversely, the largest group of genes found in three genomes were sets that excluded TM-1 (Fig. 3c).

The proximate causes of such gene PAV can be sequence evolution (for example, deletions or frameshifts) or genome annotation thresholding (for example, variable gene expression support). For example, only 454 of the 9,426 (4.8%) PAV genes between two *Panicum hallii* genomes were the result of large-effect sequence evolution, while the remainder were unannotated because of gene expression, intron structure or other non-coding sequence divergence^32^. To determine the relative contribution of coding sequence evolution to gene PAV in our four cotton genomes, we projected UA48 genes onto the other three genomes. UA48 was chosen as it has the most annotated genes. Combined, we were able to build functionally similar gene models for the majority of PAV genes (Fig. 3d), indicating that non-coding sequence evolution and annotation support are major drivers of patterns of gene presence across references. However, 3,343 genes (21.6% of PAV genes) were completely absent across the three alternative references, which supports sequence deletion and coding sequence molecular evolution as drivers for gene PAV. Combined, these results demonstrate the importance of developing cultivar-specific genomes: without the new genomes, 25,326 (8.32%, mean of 6,331 per genome) genes found within modern germplasm would have remained unidentified.

To assay the distribution of putative functional variants, we compared the three reference genomes using whole-genome alignments. We observed a small yet noteworthy set of variants between modern cultivars: relative to TM-1, we identified ‘large effect’ SNPs (for example, premature stops or loss of start codon) within 570, 558 and 610 genes in UGA230, CSX8308 and UA48, respectively. However, considering that some of these variants are shared among modern cultivars, inherited from their common ancestor, we identified 176, 119 and 184 of those genes containing large-effect SNPs unique to UGA230, CSX8308 and UA48, respectively (Supplementary Data 2).

### Interspecific introgressions impact fibre quality

**Fig. 4:**
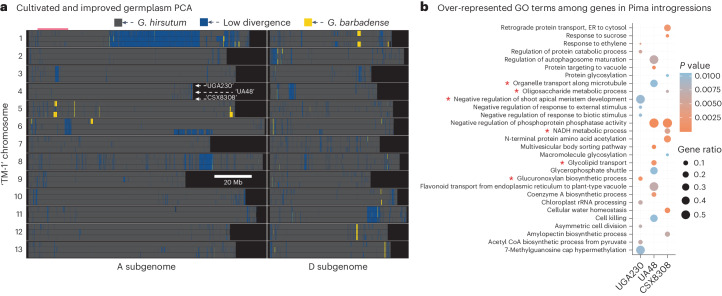
Position and transcriptional effects of Pima introgressions into modern genomes. **a**, *G. barbadense* (‘Pima’ cotton) introgressions were inferred by competitive analysis of 10 kb ‘windowed’ sequence alignments to TM-1 v3 and *G. barbadense* v1 genomes (Methods). Each white-separated row is one chromosome; columns are the two subgenomes (A subgenome, left; D subgenome, right). Within each row, the three horizontal bars represent each modern genome (from top to bottom, UGA230, UA48 and CSX8308). Dark grey regions share ancestry with TM-1, whereas yellow indicates blocks of *G. barbadense* ancestry (probably introgressions). Blue represents ‘ambiguous’; sequences where all three modern lines are ambiguous probably represent putative ancestral introgression (for example, pericentromere of Chr A01), while those found in just one reference may represent introgressions from a different Gossypium species (for example, right arm of Chr A06 in CSX8308). **b**, Top 10 GO terms (biological processes) representing an aggregate of those overrepresented (Fisher’s exact test, one-sided, *P* < 0.05) among genes within *G. barbadense* introgressed regions in each modern cultivar. Fibre-related biological processes are highlighted with an asterisk. ER, endoplasmic reticulum. Source data

**Extended Data Fig. 7:**
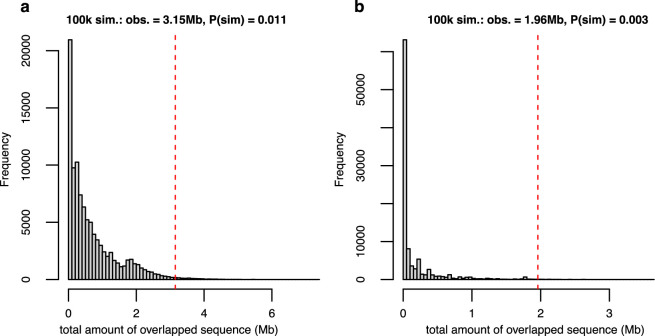
Overlap between the Pima cotton introgressed blocks identified in this study and previously observed introgression regions (Fang et al.^33^). Pima cotton introgressed regions inferred by competitive analysis of 1 kb windowed sequence alignments to TM-1 v3 and Pima v1 genomes compared with the introgressed blocks in Fang et al. ^33^. **a** 100 k simulations *P* = 0.01126 [all introgressions] (Permutation test). **b** *P* = 0.00279 [high-frequency introgressions] (Permutation test).

**Extended Data Fig. 8:**
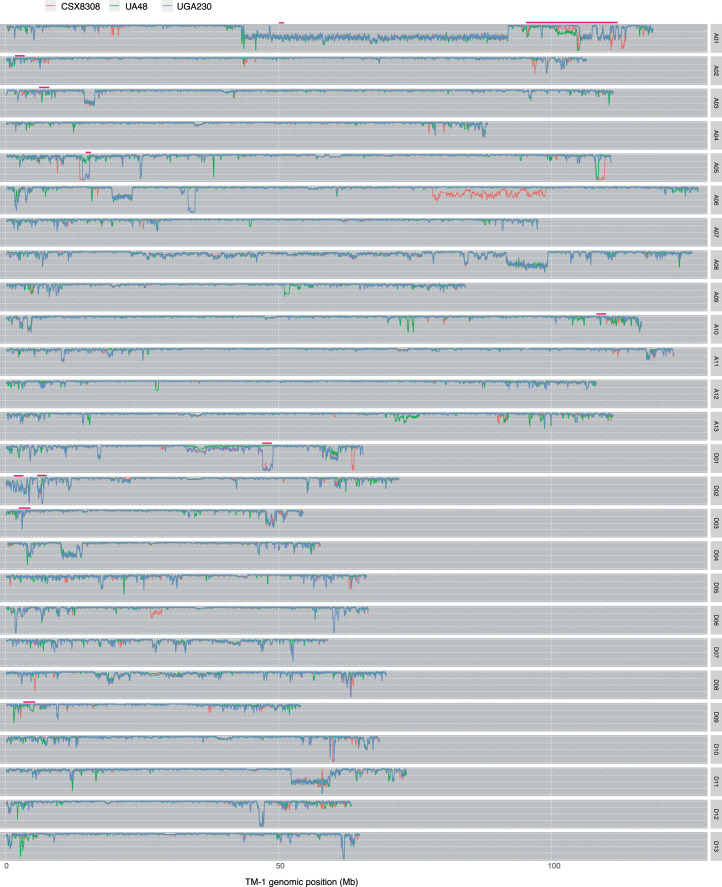
TM-1 and ‘Pima’ diverged regions. Proximate right arm of chromosome A06 (CSX8308) and a small region in CSX8308 and UA48 on the right arm of chromosome A01.

While the germplasm of modern cultivated *G. hirsutum* cotton represents a fairly recently bottlenecked gene pool, it appears that interspecific introgressions are common and variable, even within modern germplasm^33^ and especially with introgressions derived from Pima cotton (*G. barbadense*, hereon ‘Pima’). To test for the presence and frequency of introgressions, we used our highly accurate and complete assemblies and the existing Pima genome^19^. In short, we mapped 7.5 kb overlapping 10 kb genomic intervals (windows) from each cotton genome to both the Pima and TM-1 genomes and classified the alignments into three groups: (1) TM-1 mapping bias (for example, putative upland cotton), (2) Pima-biased (putative introgression), and (3) low divergence, where TM-1 and Pima have similar sequences and the modern cultivar genomes map equivalently to both. The low-divergence regions were more common than expected in the modern cultivar genomes: 148–191 Mb of the genomes mapped non-uniquely to one of the two species, which indicates putative introgressions between TM-1 and Pima. Combined, the three modern cultivar genomes harboured few (*n* = 37–51) moderately sized (50 kb to 2.05 Mb), but generally shared (Fig. 4a), regions of Pima co-ancestry, indicating that many of the introgressions occurred fairly recently and in the common ancestor of modern cultivars but not within the TM-1 lineage. As a confirmation of this approach, our introgressed blocks strongly overlapped with previously observed introgression regions^33^ (100,000 simulations *P* = 0.01126 (all introgressions) and *P* = 0.00279 (high-frequency introgressions); Extended Data Fig. 7). It is important to note that, while globally rare, there is a fourth class of alignments where TM-1 and Pima are diverged, but a modern cultivar genome does not map in a highly biased manner to either. This pattern is probably indicative of introgressions from another cotton species. Such regions are rare in non-repetitive regions of the genome; however, there are some obvious exceptions including the proximate right arm of Chr A06 (CSX8308) and a small region in CSX8308 and UA48 on the right arm of Chr A01 (Extended Data Fig. 8). These results demonstrate the scale of introgressions among cotton cultivars and further support the need for genome sequences among modern cultivars.

The fixed and polymorphic Pima introgressions offer strong a priori candidates for diverged sequences that may underlie phenotypic variation in modern germplasm. Given that Pima cotton fibre quality is the highest among cotton strains and a major goal of upland cotton breeding is to improve fibre quality, these introgressed sequences offer high-value targets for functional follow-up experiments and potential fibre quality improvement in otherwise non-modern cultivars. To infer potential phenotypic effects of the introgressions, we first verified whether the introgressed sequence is functionally active at the transcriptional level. Genes within Pima introgressions showed gene expression variation across three fibre developmental stages (7 days post-anthesis (DPA), 14 DPA and 21 DPA). An average of 36.98% (UGA230: 36.99%, UA48: 25.94%, CSX8308: 47.41%) introgressed genes showed expression variation confirming that the introgressed sequence retains some of its functional effects. Given that Pima introgressions are hypothesized to drive improved fibre quality, we also hypothesized that functional annotations among introgressed genes would be enriched in terms related to fibre development. To test this, we assessed Gene Ontology (GO) terms overrepresented among introgressed genes across modern cultivars (Fig. 4b). Enrichment was observed in processes crucial to fibre production, such as organelle transport along microtubules, oligosaccharide metabolism, glycolipid metabolism^34–36^ and biosynthesis of glucoronoxylan^37,38^. Interestingly, beyond direct fibre development, there were indications of enrichment in processes probably linked to potential domestication-associated traits such as suppression of the shoot apical meristem^39,40^.

### Leveraging modern cotton genomes for crop improvement

We used our four reference genomes to assess distinctive fibre-related biological traits within each of the modern cultivars to pinpoint promising targets that hold potential for advancing future crop enhancements. Considering the substantial resource allocation required by fibre development, a robust transcriptional response across developmental time courses was expected and observed (Supplementary Fig. 1) across all cotton lines. Differentially expressed genes showed enrichments in biological processes relevant to fibre traits in all four cotton lines (Supplementary Fig. 2). Among these genes, processes that are probable targets of selection during early domestication were identified. These include primary cell wall biogenesis, cortical microtubule organization, glucuronoxylan and lignin biosynthesis, and xylan acetylation. Primary cell wall biogenesis and cortical microtubule organization events are dynamic and highly coordinated processes. They have an essential role in aligning microtubules, providing structural support and influencing the direction of growing fibre cells^41–43^.

In addition, the biosynthesis of glucuronoxylan contributes to the construction and reinforcement of the cell wall, crucial for maintaining structural integrity during elongation, ultimately influencing strength and flexibility^37,38,44^. Moreover, lignin, a complex polymer, enhances the robustness of cell walls, elevating fibre strength and bolstering resistance against various stresses. Similarly, xylan acetylation affects the interactions between cell wall components, impacting the overall architecture and function of the cell wall, thus influencing the physical properties of the fibre^45,46^.

Genomic variations observed in modern cotton cultivars may explain some agronomic traits selected during modern breeding. For example, previous research has unveiled the role of melatonin in defence mechanisms in cotton: exogenous application of melatonin has been shown to enhance pathogen resistance, while suppressing endogenous melatonin levels compromises resistance^47^. Remarkably, the melatonin biosynthetic process is prominently represented among differentially expressed genes in CSX8308, possibly linked to its superior blight resistance^22^. In UA48, the mucilage biosynthetic process, involved in seed coat formation, water retention and influencing fibre quality^48,49^, is overrepresented. With an understanding of genes directly involved in these crucial fibre-related biological processes, the potential for impactful biotechnological interventions to enhance fibre quality and increase lint yield becomes a tangible reality.

## Discussion

Similar to many early plant reference genomes, the first allotetraploid cotton genome is a genetic standard and not a cultivar used in current breeding programmes (‘TM-1’). The development of cultivar-specific reference genomes tailored to individual breeding programmes holds potential for advancing precision genomics and enhancing the identification of trait-associated targets^50^. In this study, reference genomes for three modern cultivars that span vital breeding gene pools, along with a substantial update to the TM-1 reference genome, mark notable strides towards achieving this goal. These genomes not only capture more genetic diversity among cotton cultivars but also represent far more complete sequences of all four tetraploid cotton genomes, which will probably aid in the breeding and biotechnological improvement of cotton fibre quality and yield.

Cotton breeding efforts stand to benefit from genome-enabled methods that are not possible without reference genomes across diverse modern cultivars, such as resource-intensive fibre phenotyping and time-consuming progeny evaluations, may be expedited by selecting sequences that are only present in modern germplasm. For example, longer fibre length and improved quality are often achieved through introgressions of Pima cotton chromosomal segments^33^. Genome resources for more diverse Pima and other cotton species will improve the ability to identify and select such putative adaptive introgressions. While large introgressions can be readily identified using short-read resequencing, the same is not true for large inversions observed in this study. Long-read genotyping or potential imputations through pan-genome reconstruction could pave the way for structural variation diagnoses across the breeding pedigrees of cotton.

Despite the advances our new genomes present, there remains room for additional multi-reference-enabled breeding and diversity discovery in cotton and its wild relatives. We envision that reference genomes will soon be available for more genotypes of upland cotton and other *Gossypium* species. Expanding the phylogenetic distribution of genome resources, and crucially the traits and climatic regions that accompany reference genomes, will enable improved modelling of genotype–environment–trait interactions. The resulting candidate sequences and markers will let breeders rapidly adapt cotton germplasm to novel and changing environmental pressures. These future resources will complement the analyses presented here and allow for causal inference between introgressions, genetic diversity and agronomic traits.

In species characterized by limited genetic diversity, gene PAV and CNV may be valuable trait-associated molecular targets. Our genomes and analyses demonstrate considerable PAV among the three sequenced modern cultivars and the genetic standard TM-1. Notably, the presence of the highest number of private gene sets in TM-1 underscores its phylogenetically divergent position relative to modern cultivars while also showing genes unique to, and at high frequency within, modern germplasm. In addition to PAV, large-scale sequence and structural variations represent crucial sources of heritable trait diversity and potential targets for enhancement through selective breeding. The identification of inversions, translocations and duplications within these highly collinear cotton genotypes, as catalogued in our study, offers a genomic solution to accelerate breeding strategies. Together, the high-quality reference genomes and the results of our comparative genomic analyses of modern germplasm hold promise for advancing both functional genomics and breeding efforts. These advancements bring us one step closer to capitalizing the potential of genomic breeding and genome editing for improving cotton fibre quality and yield as well as crop resilience.

## Methods

### Sequenced genotypes

*G. hirsutum* L. acc. TM-1 (1008001.06), UGA230, UA48 and CSX8308 were grown in a greenhouse at Clemson University. Young leaves were collected for high-molecular-weight DNA extraction using a published method^51^.

TM-1 was derived from Deltapine 14 and inbred for multiple generations^16^. The stocks have been maintained at the Southern Plains Agricultural Research Center, USDA, with seeds distributed among different laboratories, which may have resulted in 4–6 genotypes that are collectively known as similar TM-1 offspring.

Cultivar ‘UGA230’ (PVP 201500309 or UGA 2004230) is a conventional upland cotton cultivar that was developed and released by the University of Georgia Agricultural Experiment Station in 2009. UGA230 is typical in appearance with normal leaf shape and colour. Flowers have cream-coloured petals without petal spots and cream-coloured pollen. Vegetative branches (monopodia) are found on the lower plant with fruiting branches (sympodia) found on the vegetative branches. Higher on the plant on the main axis without clustering, it has nectaries and gossypol glands. Developed from a cross between PD94045 X and DPX8C80, UGA230 has high yield potential with broad adaptation, particularly to regions with long growing seasons such as the Southeastern US Cotton Belt. In addition, UGA230 has an excellent fibre quality package. For example, it had the longest fibre length (upper half mean) compared with the most popular commercial cultivars at the time of its release. Other fibre quality measures that are considered most important (strength, fineness and uniformity of length) were also very competitive. UGA230 has made a tremendous impact on modern US cotton germplasm, serving as a parental line in many public and private breeding programmes.

Cultivar ‘UA48’ (registration number CV-129, PI 660508) is a conventional upland cotton cultivar that was released by the Arkansas Agricultural Experiment Station in November 2010^23^. The parent lines of UA48 are Arkot 8712 (ref. ^52^) and FM 966 (PVP 200100209). UA48 was released as part of an ongoing effort to develop genotypes with enhanced yield, yield components, earliness, host plant resistance and fibre properties. In most tests, UA48 produced lint yield comparable to ‘DP 393’, a well-adapted conventional cultivar. UA48 is best adapted to silt loam soils in the northern areas of US cotton production. UA48 matures as early as any cultivar that is adapted to the Mississippi River Delta. It shows high resistance to bacterial blight and performs equally well as DP 393 against other diseases. The fibre quality of UA48 is exceptional. In most tests, its fibre length, uniformity and strength exceeded most, and frequently all, other entries. Its micronaire value is higher than that of DP 393. UA48 shows an unusual combination of high yielding ability, early maturity and high fibre quality.

Cultivar ‘CSX8308’ (Siokra 250) was developed in a planned breeding programme at CSIRO Australia by crossing two proprietary breeding lines 64005-56OL × 64014-338NL. It is an okra-leaf variety with broad adaptability across Australian cotton-growing regions and shows resistance to bacterial blight and has high yield and very high gin turnout with an excellent combination of fibre quality traits. During selection, specific emphasis was placed on resistance to the Australian biotype of fusarium wilt. It is a medium stature line with medium-late crop maturity.

### Plant growth and RNA extraction

Cotton plants were grown in 3-gallon pots (3 pots for each genotype). Five seeds per pot were sown in 3B soil (Fafard 3-B Mix, Fafard) containing 1 teaspoon of fertilizer (Osmocote 18-6-12), covered with around 0.5 inches of germination mix and kept in a greenhouse for 6 days. After thinning, only one seedling in each pot with similar status was kept and grown under greenhouse conditions (natural light with 16-h photoperiod supplemental illumination at 30 °C/25 °C in light/dark). The three plants for each genotype were developmentally synchronized to flowering where four flowers were bagged and tagged to ensure self-pollination. DPA were determined when the bagged flowers fully bloomed in the morning. Cotton bolls were collected at 7 DPA, 14 DPA and 21 DPA, and fibres were carefully separated from other tissues, blot dried using Kimwipes, weighed, packaged in aluminium foil, snap-frozen in liquid nitrogen and stored at −80 °C before nucleic acid extraction. Total RNA was isolated using LiCl precipitation methods described previously^53^. RNA purity was verified with ultravoilet spectroscopy (NanoDrop 8000) and integrity validated using an Agilent 2100 RNA bioanalyser.

### Histology preparation and scanning electron microscopy

Mature fibres were harvested from each plant and dried for at least 10 days before embedding. Several hundred fibres were combed straight, twisted into bundles and inserted in Simport M510-2 SLIMSETT cassettes and trimmed to fit the mould to avoid folding. The samples were embedded in type L paraffin using a Tissue TE-II embedding station and allowed to solidify overnight. The next day, each sample was cut to a thickness of 10 μm using a Leica RM2165 microtome. Microtomed sections were then placed in a hot bath at 37 °C followed by mounting on tanner adhesive glass slides. Slides were incubated on a hot plate at 28 °C overnight and deparaffinized the next day by performing three washes in xylene, two washes in 100% ethanol, two washes in 95% ethanol, followed by three rinses in distilled water. All washes lasted 2 min. Samples were sputter-coated with platinum using a Hummer 6.2, and images were collected with a Hitachi SU6600 or Hitachi SU5000 field emission electron microscope at an acceleration voltage of 3 kV. All images were captured at ×1,000 magnification at a resolution of 1,280 × 960. Scaled images were analysed using ImageJ (v.1.54c)^54^ by first setting the scale to match the image at 10 pixels per 1 μm. The freehand selection tool was then used to outline the perimeters of the primary cell wall and the internal lumen. Data were moved from the native format in ImageJ to a tabular file for analysis with JMP (v.16.2). Once imported to JMP, each variable (external circumference, internal area, lumen circumference, lumen area and lumen area/internal area) was compared among genotypes using an analysis of variance (ANOVA) and Tukey’s honestly significant difference test to determine statistical significance of the differences.

### Morphological and yield metrics

To assess fibre traits of selected modern cultivars, we collected fibre quality and yield metrics in nine different locations in the USA. We measured lint per cent, lint yield, oil per cent, protein per cent, staple length or upper half mean length, uniformity index, strength, micronaire, fibre elongation and seed index. A mixed model analysis considering cultivar genotypes (*G*) as fixed effects and environments (*E*; year and location combination), replications within the environment and the *G* × *E* interaction as random effects showed that genotypic effects for all traits were statistically significant (Supplementary Data 3).

ANOVA was carried out using mixed model analysis in RGxE (v.1)^55^, an R program for genotype by environment interaction analysis, using the lmer function from the lme4 (v.1.1-32)^56^ package. Cultivar genotypes (*G*) were considered fixed effects, and the environments (*E*; year and location combination), replications within the environment and the *G* × *E* interaction were considered random effects. For the fixed effects, *P* values were computed using *F* ratio tests with the Kenward–Rogers (KR) approximation for degrees of freedom, and *P* values for the random effects were generated using likelihood ratio tests following model comparisons and ANOVA. Least squares means for the mixed models were computed using lsmeans (v.2.30-1)^57^ (Supplementary Data 3).

### Genome sequencing and assembly

For de novo assembly of TM-1, UGA230, UA48 and CSX8308, sequencing was performed using Pacific Biosciences (PacBio) SEQUEL II, Illumina NovaSeq and Hi-C sequencing technologies. The TM-1 v3 genome was assembled using MECAT (v.1.2)^58^ with 116.73× PacBio sequence coverage, and the resulting assembly was polished using ARROW (v.2.2.2)^59^. Misjoins in the assembly were identified using Hi-C (Supplementary Fig. 3) and 108,262 unique, non-repetitive, non-overlapping 1 kb sequences that were extracted from the existing *G. hirsutum* TM-1 v2 assembly^19^ and aligned to the polished TM-1 v3 assembly. Three misjoins were identified in the polished assembly. The misjoin-resolved contigs were then oriented, ordered and joined together with the aforementioned 1 kb sequences as syntenic markers. A total of 212 joins were applied to the assembly to form the final assembly consisting of 26 chromosomes. Each chromosome join was padded with 10,000 Ns. Adjacent redundant sequences were identified on the joined contig set. Redundant flanking regions on gaps were collapsed using the longest common substring between the two haplotypes. In total, 116 adjacent redundant sequences were collapsed. Finally, contigs from TM-1 v2 were used to patch 31 remaining gaps in the TM-1 v3 assembly. The remaining scaffolds were screened for bacterial proteins and organelle sequences using the GenBank non-redundant database, and identified contaminants were removed. Homozygous SNPs and indels were corrected in the release consensus sequence using 55× Illumina reads (2 × 150, 400 bp insert) by aligning the Illumina reads using BWA-MEM (v.0.7.17)^60^ and identifying homozygous SNPs and indels with GATK’s UnifiedGenotyper tool (v.4.3.0.0)^61^. A total of 438 homozygous SNPs and 11,313 homozygous indels were corrected in the release. The final TM-1 v3 reference genome contains 2,277.5 Mb of sequence, consisting of 91 contigs with a contig N50 of 40.0 Mb and 99.4% of the bases assembled into 26 chromosomes.

UGA230, UA48 and CSX8308 genomes were assembled in an identical manner to TM-1 using 108,262 unique, non-repetitive, non-overlapping 1 kb sequences extracted from the TM-1 v2 assembly as syntenic markers. Assembly and polishing were conducted following TM-1 v3 genome with PacBio coverage (95.5×/93.7×/114.46×, UGA230/UA48/CSX8308, respectively); 8/56/14 misjoins and 293/933/296 contig joins were identified with Hi-C (Supplementary Figs. 3 and 4) and syntenic markers. A total of 118/321/112 alternative haplotypes were collapsed, and 154/1,018/138 homozygous SNPs and 7,243/91,504/22,167 homozygous indels were corrected using Illumina reads. The UGA230 genome contained 2,274.6 Mb of sequence in scaffolds with a contig and scaffold N50 of 27.4 Mb and 107 Mb, respectively, and 99.5% of bases assembled into 26 chromosomes. The UA48 genome contained 2,289.0 Mb of sequence in scaffolds with a contig and scaffold N50 of 7.8 Mb and 105.8 Mb, respectively, and 98.2% of bases assembled into 26 chromosomes. The CSX8308 genome contained 2,276.1 Mb of sequence in scaffolds with a contig and scaffold N50 of 29.9 Mb and 107.2 Mb, respectively, and 99.6% of bases assembled into 26 chromosomes.

In all genomes, contigs containing telomeric sequences were identified using the (TTTAGGG)_*n*_ repeat, and care was taken to ensure that contigs terminating in this sequence were properly oriented in the production assembly.

It is important to note that biology plays an important role in the genome assembly size and contiguity. For example, UA48 is nearly two orders of magnitude more heterozygous than the other sequenced genotypes: it has 525 heterozygous bases per Mb compared with 6 per Mb in TM-1. Partially inbred pedigrees such as that of UA48 have long runs of homozygosity due to identity-by-descent punctuated by patches of high heterozygosity. Representing such a genome as haploid requires selecting between two haplotypes in each heterozygous region. Our genome assembly approach chooses the longer of the two meiotic homologous contigs in heterozygous regions, then resolves potentially duplicated sequences at the contig end joins. Choosing the longer contig is necessary to avoid gaps where one haplotype does not extend fully through a heterozygous block. However, it also produces a slightly larger genome size, which may introduce some ‘redundancy’. For example, if two biological haplotypes in a heterozygous region differ in the copy number of a tandem array (and the longer contig has a higher copy number), the contig with more copies will be preferentially retained. This is still a biologically accurate representation of the sequence but also increases redundancy by representing the longer and higher copy array. This heterozygosity yields a UA48 assembly with more gaps in repeat regions. As such, it is not surprising that UA48 has ~11 Mb less sequence in the chromosomes but has 7.8 Mb more repetitive sequence in the bottom drawer than TM-1 v3.

### Genome annotation

Genome annotation was accomplished using our standard pipeline developed by the Department of Energy’s Joint Genome Institute and Phytozome. To build the annotations, first transcript assemblies were made from 5.47 billion pairs of 150 bp stranded paired-end Illumina RNA sequencing (RNA-seq) reads (Supplementary Data 4) using PERTRAN (details of which have previously been published^32^). In brief, PERTRAN conducts genome-guided transcriptome short-read assembly via GSNAP (v.2013-09-30)^62^ and builds splice alignment graphs after alignment validation, realignment and correction. Subsequently, 289,675, 343,308, 348,112 and 345,206 transcript assemblies were constructed for TM-1, UGA230, UA38 and CSX8308, respectively, using PASA (v.2.0.2)^63^ from RNA-seq reads. Loci were determined by EXONERATE (v.2.4.0)^64^ alignments of cotton genome transcript assemblies and proteins from *Arabidopsis thaliana*^65^, soybean^66^, Nipponbare rice^67^, *Setaria viridis*^68^, *Sorghum bicolor*^69^, *Theobroma cacao*^70^, grape^71^ and Swiss-Prot^72^ proteomes. These alignments were accomplished against repeat-soft-masked genomes using RepeatMasker (v.4.1.3)^73^ (repeat library from RepeatModeler (v.open1.0.11) and RepBase^74^) with up to 2,000 bp extension on both ends unless extending into another locus on the same strand. Incomplete gene models, which had low homology support without full transcriptome support, or short single-exon genes (<300 bp coding DNA sequences) without protein domains or good expression were removed.

### Identification of centromeres and telomeres

To identify centromeres, we extracted 25-mers from putative centromeric regions determined previously^75^ and subtracted any that occurred less than 25 times in the centromere or were found in non-centromeric regions in the TM-1 v2.1 (ZJU_TM1) genome^75^. There were 3,039,983 of these ‘diagnostic’ 25-mers. Fifth quantile of the minimum peak density of these kmers in the ZJU_TM1 genome was 2.04%; as such, we define centromeres in our genomes as any region where the diagnostic kmers cover ≥2.04% of overlapping 250 kb blocks of 50 kb sequence. Telomeres were identified using the find_telomeres function in the GENESPACE (v.1.3.1)^28^ with CCCGAAA, CCCTAAA, TTTCGGG and TTTAGGG as putative telomeric kmers (Supplementary Fig. 5 and Supplementary Data 5).

### RNA-seq library construction and sequencing

Tissue was ground under liquid nitrogen and kept at −80 °C until use. High-quality RNA was extracted using standard Trizol-reagent-based extraction^76^. The integrity and concentration of RNA preparations were initially checked using a Nano-Drop ND-1000 (Nano-Drop Technologies) and then by a bioanalyser (Agilent Technologies). Plate-based RNA sample preparation was performed using the PerkinElmer Sciclone NGS robotic liquid handling system using Illumina’s TruSeq Stranded mRNA HT sample prep kit utilizing poly-A selection of messenger RNA following the protocol outlined by Illumina under following conditions: total RNA starting material was 1 μg per sample and 8 cycles of PCR were used for library amplification. The prepared libraries were then quantified by qPCR using the Kapa SYBR Fast Illumina Library Quantification Kit (Kapa Biosystems) and run on a Roche LightCycler 480 real-time PCR instrument. The quantified libraries were then prepared for sequencing on the Illumina HiSeq sequencing platform utilizing a TruSeq paired-end cluster kit, v4, and Illumina’s cBot instrument to generate a clustered flow cell. Sequencing of the flow cell was performed on an Illumina HiSeq2500 sequencer using a HiSeq TruSeq SBS sequencing kit, v4, following a 2 × 150 indexed run recipe. The same standardized protocols were used to prevent any batch effects among samples throughout the project.

### Gene expression analysis

Illumina paired-end RNA-seq 150-bp reads were quality trimmed (*Q* ≥ 25), and reads shorter than 50 bp after trimming were discarded. High-quality sequences were aligned to reference genomes using STAR (v.2.7.8a)^77^, and the counts of reads uniquely mapping to annotated genes were obtained using featureCounts, part of the Rsubread package (v.2.12.3)^78^. Fragments per kilobase of exon per million fragments mapped and transcripts per million values were calculated for each gene by normalizing the read count data to both the length of the gene and the total number of mapped reads in the sample, and the metric was considered for estimating gene expression levels^79,80^. Genes with low expression were filtered out by requiring ≥2 relative log expression normalized counts in at least two samples for each gene. Differential expression analysis was conducted using a Wald test in DESeq2 (v.1.30.1)^81^ with an adjusted *P*-value threshold of <0.05 using the Benjamini and Hochberg method and a log_2_ fold change >1 as the statistical cut-off for differentially expressed genes.

### GO and KEGG pathway enrichment analysis

GO enrichment analysis of differentially expressed genes, expanded gene families and genes within Pima cotton introgressed regions was performed using topGO (v.2.42.0)^82^, an R Bioconductor package, to determine overrepresented GO categories across biological process, cellular component and molecular function domains. Enrichment of GO terms was tested using Fisher’s exact test with *P* < 0.05 considered significant. KEGG^83^ pathway enrichment analysis was also performed on these gene sets based on hypergeometric distribution tests, and pathways with *P* < 0.05 were considered enriched.

### Comparative genomics

GENESPACE^28^ was used to identify orthologous genes, understand the scale of synteny between cotton genomes, infer gene PSV and generate pan-gene sets. Orthologous groups among reference genomes were identified using OrthoFinder^84^ based on all annotated protein-coding sequences. GENESPACE then integrated the orthologous gene pairs into collinear blocks, which effectively masked paralogous regions, thus permitting higher confidence visualizations and interpretations. Depending on how the OrthoFinder run was parameterized, homeologous regions were either flagged as paralogous and excluded (if only tetraploid cotton genomes were used) or included in orthologous gene clusters (if subgenomes were split or a diploid outgroup was included). These orthogroups were integrated with PLAZA^30^ gene families and assessed for gene family expansions and contractions between genomes.

We compared sequence similarity and positional mapping using minimap2 (v.2.26)^85^ alignments between 7.5 kb overlapping 10 kb fragments (‘windows’) of the query genome against the reference genome with the following parameters: optimized for closely related genome assemblies (‘preset’ asm5), no secondary hits, kmer word size of 25 and minimizer window size of 20. The resulting mapping (.paf) file for each comparison (see below) was subset to only the highest-confidence hits by (1) retaining the single best hit per query (‘nhits’ = 1), (2) excluding alignments with pairwise differences >2% (‘pid’ = 0.98), (3) excluding alignments covering <75% of the query (‘pcov’ = 0.75) and (4) pruning to collinear hits via GENESPACE^28^ with block size of 5 hits and a window of 10 hits. These mappings were used for four distinct analyses (with modifications). (1) synteny map: defining a single coordinate system across the four *G. hirsutum* genomes (reference) so that genome-specific information can be projected across all genomes; (2) tests for regions of low divergence: *G. hirsutum* (reference) to *G. barbadense* (query, preset = asm10, pid = 0.99); (3) test for introgressions: mapping of each *G. hirsutum* genome (query) to a concatenated *G. hirsutum* and *G. barbadense* reference (pid = 0.99, 2.5 kb overlapping windows); (4) subgenome synteny: subgenome A–D synteny (preset = asm20, pid = 0.75).

These windowed genome alignments were used in three ways. First, a subgenome A–D map was built by clustering the rank-order transformed positions of between-subgenome hits using dbscan (v.1.1-11)^86^. This was then plotted using GENESPACE riparian plotting subroutines. Second, we built a common coarse-scale coordinate system between the three modern references, TM-1 and *G. barbadense*, where uniquely mappable 10 kb fragment positions can be tracked across all five genomes. Finally, we used the common coordinate system to map divergence and interspecific introgressions across the cotton genomes. To accomplish this, we first defined regions of low divergence between all four *G. hirsutum* genomes and *G. barbadense* as 200 kb intervals where >50% of the 5 kb overlapping 10 kb intervals had >98% similarity. We then extracted the best competitive mappings for each trio (*G. hirsutum* 1: (*G. hirsutum* 2 – *G. barbadense*)) for all windows that did not overlap low divergence regions between *G. hirsutum* 2 and *G. barbadense*. These mappings were converted into introgression blocks where ≥10 consecutive windows in (*G. hirsutum* TM-1) uniquely mapped to *G. barbadense* chromosomes in the concatenated genome. For each 49-window overlapping 50-window interval, we calculated ‘%*G. barbadense*’ as the percentage of windows that mapped with a higher score to *G. barbadense* than to *G. hirsutum*. Intervals where %*G. barbadense* ≥ 70% were the introgressed sequences. Intervals with 30 > %*G. barbadense* < 70 were ambiguous. For visualization and analysis purposes, the introgression coordinates were projected back onto the TM-1 reference using the synteny map described above. Introgression blocks were plotted with ggplot2 (v.3.4.2)^87^. Data processing and organization were accomplished with data.table (v.1.14.8)^88^.

It is important to note that we used the JGI v2 TM-1 reference for most comparisons of legacy genomes instead of the Huang reference. Despite its higher level of contiguity, the Huang reference used a qualitatively different annotation method, which is not directly comparable with the JGI annotation methods, which integrates homology and gene structure modelling with evidence from flcDNA and RNA-seq methods. As such, v2 provided a more comparable baseline for comparative genomics studies.

### Large structural variation analysis

Pairwise combinations of reference genome assemblies were aligned using minimap2 (v.2.26) with the parameter setting ‘–ax asm5 -eqx’. The resulting alignments were used to identify structural rearrangements and local variations using SyRI (v.1.6.3)^89^ and visualized with plotsr (v.1.1.0)^90^.

To confirm the presence of large structural variations identified within genomes, we performed reciprocal mapping of Hi-C data using the Juicer (v.1.6)^91^ pipeline. Specifically, Hi-C libraries from both TM-1 and CSX8308 were mapped to the TM-1 reference to pinpoint structural variations specific to CSX8308 compared with TM-1. The Hi-C contact maps were visualized using JuiceBox (v.2.15)^92^.

### Variant calling

The cotton resequencing samples from ref. ^93^ were aligned to TM-1 v3, and SNPs were called using BWA-MEM (v.0.7.17). The resulting bam file was filtered for duplicates using Picard (v.2.27.5) (http://broadinstitute.github.io/picard). A GVCF was created for each sample using SAMtools mpileup (v.1.17)^94^ and Varscan (v.2.4.0)^95^ with a minimum coverage of eight and a minimum alternate allele count of four. SNPs within annotated repeat regions were removed from further analyses. Only SNPs with ≤20% missing data and minor allele frequencies >0.005 were retained. The 400 genotypes we selected were chosen owing to their diverse positions in the genetic structure of cultivated cotton out of a larger set of ~1,500 samples^93^. Specifically, we selected the majority of the ‘Ghlandrace’ accessions (218 of 256) and a notable set of diversity (228) from within the US and Chinese ‘improved’ cultivars. Given the topology of Li’s clustering tree, these samples should cover the vast majority of variation explored therein.

### Population structure

Population structure for SNP was estimated using fastStructure (v.1.0)^96^. SNP markers were randomly subsetted to 50,000 by linkage disequilibrium pruning (parameters: –indep-pairwise 50 50 0.5) using plink (v.1.9)^97^. A sample with a maximum membership coefficient (qi) of <0.7 was considered admixed. Only non-admixed samples from the SNP analysis were used for further population genomics analysis. For SNP markers, multidimensional scaling, identity by state and linkage disequilibrium estimates (parameters: –r2–ld-window-kb 500 –ld-window-r2 0) were performed using plink.

### Reporting summary

Further information on research design is available in the Nature Portfolio Reporting Summary linked to this article.

### Supplementary information

Supplementary Information Supplementary Figs. 1–5.

Reporting Summary

Supplementary Data 1 Gene synteny map between *G. hirsutum* TM-1 versions v3 and v2.

Supplementary Data 2 TM-1 gene lists with large-effect SNPs relative to modern cotton cultivars.

Supplementary Data 3 Statistical results demonstrating the significance of both the fixed effects of cotton cultivars and the random effects within the model among fibre biometrics.

Supplementary Data 4 RNA-seq samples generated and analysed in this study.

Supplementary Data 5 Physical positions of centromeres and telomeres in TM-1, modern cotton cultivars and ZJU_TM1 chromosomes.

### Source data

Source Data Fig. 4 Gene Ontology terms overrepresented among genes within *G. barbadense* introgressed regions in each modern cultivar.

Source Data Extended Data Fig./Table 1 Repeat and gene content comparison between TM-1 reference genome versions, v2 and v3.

Source Data Extended Data Fig./Table 2 Fibre quality and cotton yield data.

Source Data Extended Data Fig. 6 Gene Ontology terms overrepresented among specific expanded gene families of modern cultivar lines.

## Figures and Tables

**Extended Data Table 1:**
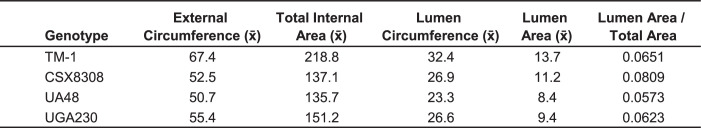
Average measurements of cotton fibre components

Measurements include external circumference, total internal area, lumen circumference, and lumen area.

**Extended Data Table 2:**
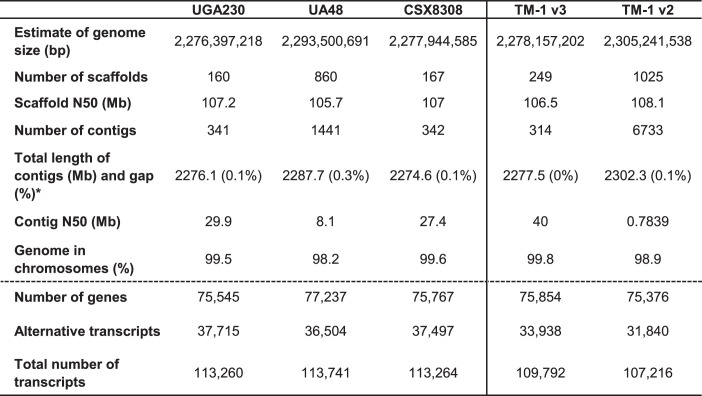
Genome assembly and annotation statistics for three modern cotton cultivars and TM-1

## Data Availability

Reference genome assembly and annotation files of TM-1 (v.3.1), UGA230 (v.1.1), UA48 (v.1.1) and CSX8308 (v.1.1) genomes are available at https://phytozome-next.jgi.doe.gov/. All raw sequence reads have been deposited in the NCBI SRA database under BioProject accessions PRJNA1071074, PRJNA1071075, PRJNA1071076 and PRJNA1071077. Source data are provided with this paper.
