# Supplementary material for: Genome resources for three modern cotton lines guide future breeding efforts
Source: Nat Plants. 2024 May 30;10(6):1039–51. doi: 10.1038/s41477-024-01713-z (PMC11208153; doi:10.1038/s41477-024-01713-z)
Supplement: Supplementary file 1 — Supplementary Figs. 1–5. [file 41477_2024_1713_MOESM1_ESM.pdf]

nature plants

Supplementary information https://doi.org/10.1038/s41477-024-01713-z

Genome resources for three modern cotton lines guide future breeding efforts

In the format provided by the authors and unedited

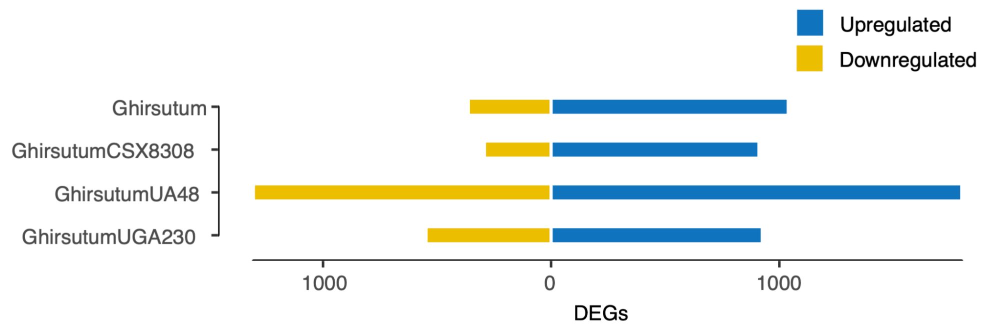

Supplementary Figure 1 | Transcriptomic response during fiber developmental stages. Numbers of differentially expressed genes (Wald test, Benjamini-Hochberg adjusted *P* <1e-100) across three fiber developmental stages (7, 14 and 21 days post anthesis, DPA) in TM-1, CSX8308, UGA230 and UA48.

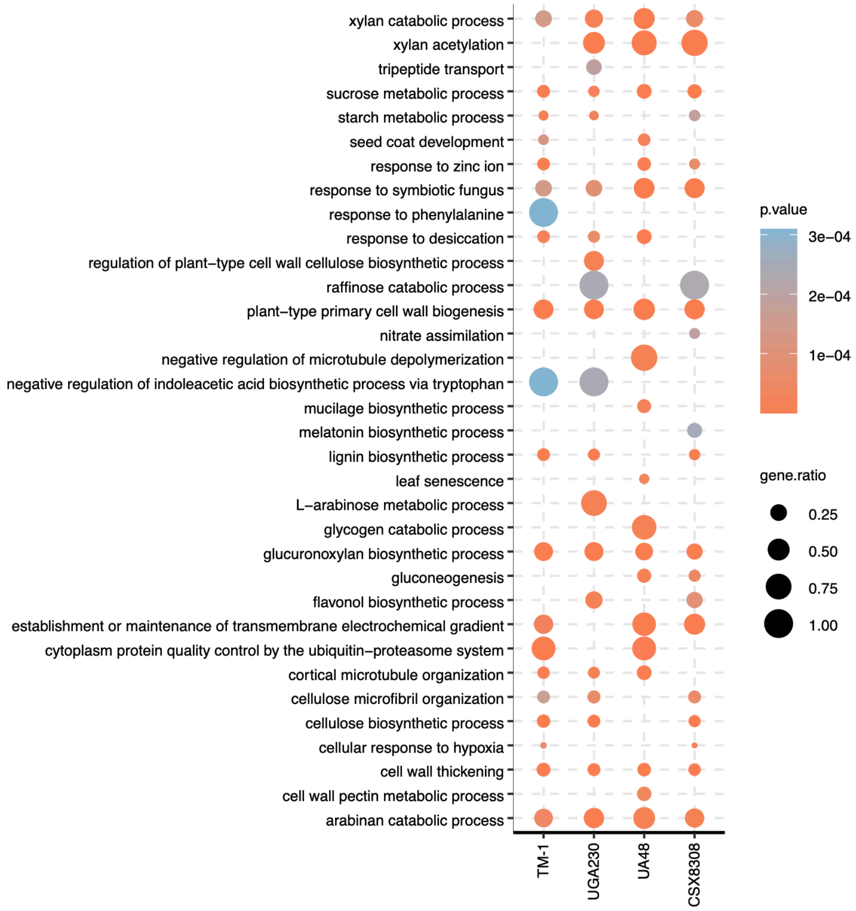

Supplementary Figure 2 | Overrepresented Gene Ontology terms (biological processes). Top 20 significant Gene Ontology terms (Fisher's exact test, one-sided, *P* <0.05) overrepresented among differentially expressed genes identified across fiber time course in TM-1 and modern cotton lines.

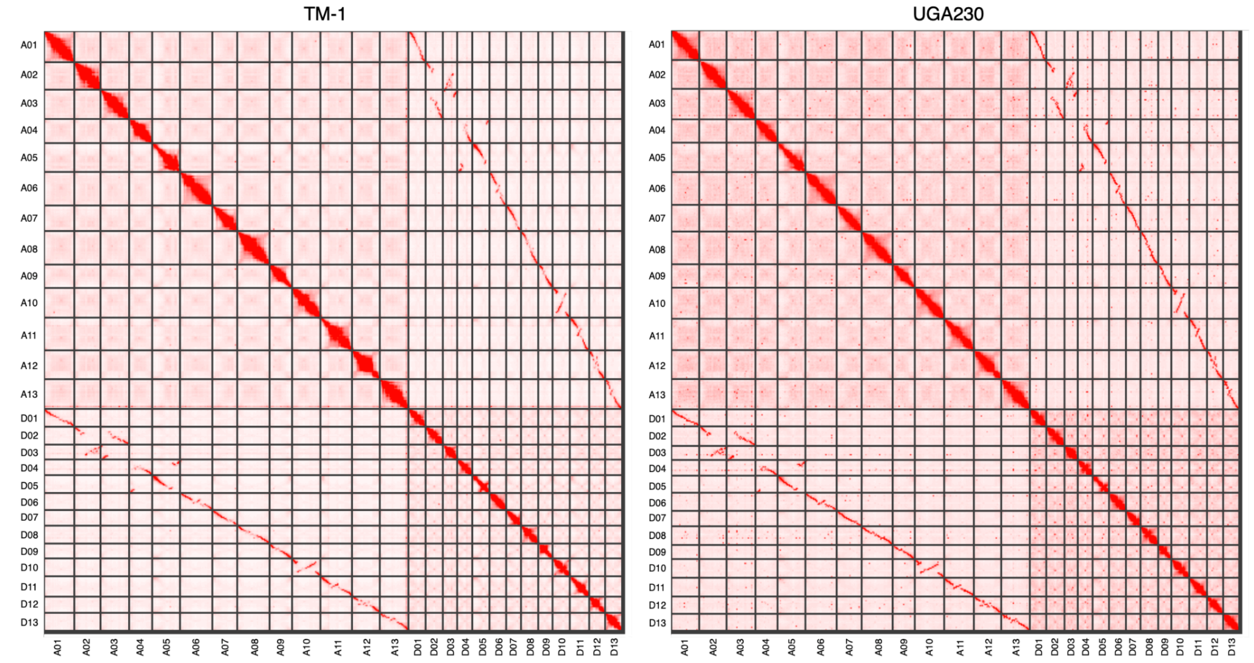

Supplementary Figure 3 | Hi-C interaction heatmap of the TM-1 and UGA230 genomes. Hi-C contact data mapped against TM-1 (left) and UGA230 (right) reference genomes. Robust signals along the diagonal suggest precise contigs orientation on the pseudo-chromosomes.

.

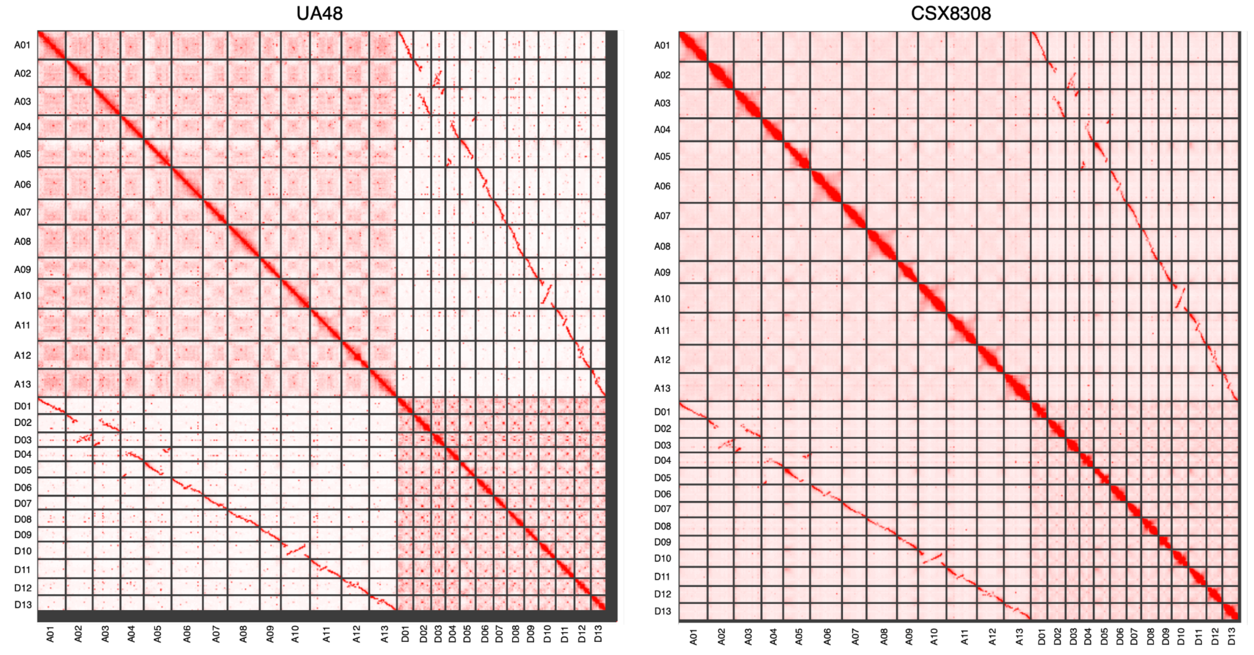

Supplementary Figure 4 | Hi-C interaction heatmap of the UA48 and CSX8308 genomes. Hi-C contact data mapped against UA48 (left) and CSX8308 (right) reference genomes. Robust signals along the diagonal suggest precise contigs orientation on the pseudo-chromosomes.

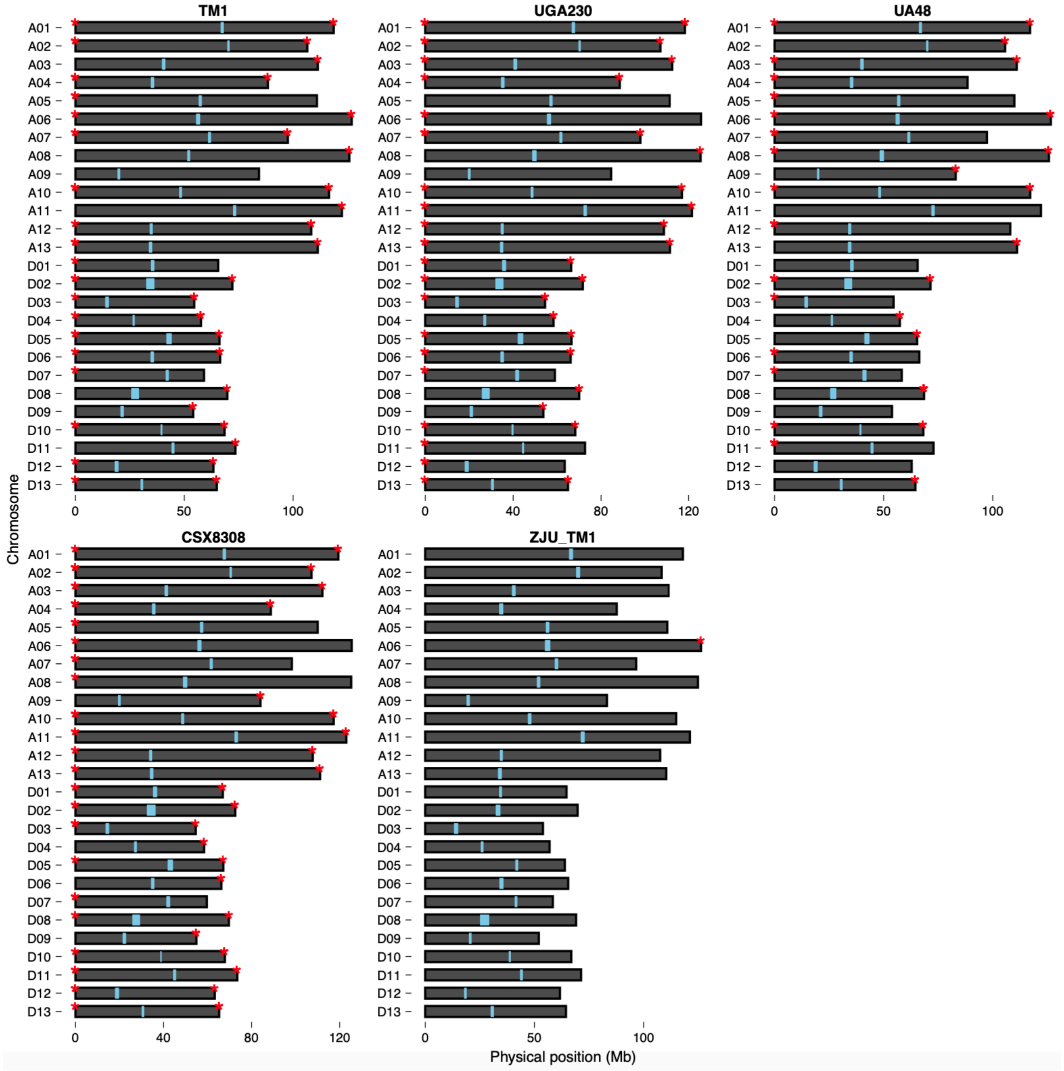

Supplementary Figure 5 | Centromeres and telomeres in TM-1, modern cotton lines (CSX8308, UA48 and UGA230) and ZJU_TM1 (Hu et al., 2019). Graphical representation of the genomic positions of centromeres (blue blocks) and telomeres (red *) on 26 chromosomes.
