# Supplementary material for: Genome resources for three modern cotton lines guide future breeding efforts
Source: Nat Plants. 2024 May 30;10(6):1039–51. doi: 10.1038/s41477-024-01713-z (PMC11208153; doi:10.1038/s41477-024-01713-z)
Supplement: Supplementary file 2 — Reporting Summary [file 41477_2024_1713_MOESM2_ESM.pdf]

nature portfolio

Corresponding author(s): Avinash Sreedasyam

Last updated by author(s): Apr 23, 2024

Reporting Summary

Nature Portfolio wishes to improve the reproducibility of the work that we publish. This form provides structure for consistency and transparency in reporting. For further information on Nature Portfolio policies, see our Editorial Policies and the Editorial Policy Checklist.

Statistics

For all statistical analyses, confirm that the following items are present in the figure legend, table legend, main text, or Methods section.

n/a	Confirmed	
☐	☒	The exact sample size (*n*) for each experimental group/condition, given as a discrete number and unit of measurement
☐	☒	A statement on whether measurements were taken from distinct samples or whether the same sample was measured repeatedly
☐	☒	The statistical test(s) used AND whether they are one- or two-sided<br>*Only common tests should be described solely by name; describe more complex techniques in the Methods section.*
☒	☐	A description of all covariates tested
☐	☒	A description of any assumptions or corrections, such as tests of normality and adjustment for multiple comparisons
☐	☒	A full description of the statistical parameters including central tendency (e.g. means) or other basic estimates (e.g. regression coefficient) AND variation (e.g. standard deviation) or associated estimates of uncertainty (e.g. confidence intervals)
☐	☒	For null hypothesis testing, the test statistic (e.g. *F*, *t*, *r*) with confidence intervals, effect sizes, degrees of freedom and *P* value noted<br>*Give P values as exact values whenever suitable.*
☒	☐	For Bayesian analysis, information on the choice of priors and Markov chain Monte Carlo settings
☒	☐	For hierarchical and complex designs, identification of the appropriate level for tests and full reporting of outcomes
☒	☐	Estimates of effect sizes (e.g. Cohen's *d*, Pearson's *r*), indicating how they were calculated

Our web collection on statistics for biologists contains articles on many of the points above.

Software and code

Policy information about availability of computer code

Data collection	DNA and RNA data were collected via Illumina NovSeq6000 and PacBio Sequel II internal routines. All phenotype data was collected and input manually using the best practices for fiber quality and yield metrics.
Data analysis	All data analysis was conducted through programs described in the methods. The majority of which was accomplished in the R (v4.2.2) environment for statistical computing.<br><br>Genome Assembly: MECAT (v.1.2), ARROW (v.2.2.2), Juicebox (v2.15), Juicer (v1.6), BWA-MEM (v0.7.17), GATK (v4.3.0.0);<br>Histology: ImageJ (v.1.54c), JMP (v16.2);<br>Yield metrics: RGxE (v.1), lme4 (v.1.1-32), lsmeans (v.2.30-1);<br>Genome Annotation: GSNAP (v2013-09-30), PASA (v2.0.2), EXONERATE (v2.4.0), RepeatModeler (v.open1.0.11), Repeatmasker (v4.1.3), FGENESH+(v3.1.0), AUGUSTUS (v3.1.0), BUSCO (v5.5);<br>Comparative Genomics and visualization: GENESPACE (v1.3.1), Orthofinder (v2.5.4), MCScanX (v2), SyRI (v1.6.3), Biostrings (v2.70.2), minimap2 (v2.26), dbscan (v1.1-11), data.table (v1.14.8), SAMtools (v1.17), Varscan (v2.4.0), fastStructure (v1.0), plink (v1.9), Picard (2.27.5); ggplot2 (v3.4.2);<br>Gene expression analysis: STAR (v2.7.8a), Rsubread (v2.12.3), DESeq2 (v1.30.1), topGO (v.2.42.0);

For manuscripts utilizing custom algorithms or software that are central to the research but not yet described in published literature, software must be made available to editors and reviewers. We strongly encourage code deposition in a community repository (e.g. GitHub). See the Nature Portfolio guidelines for submitting code & software for further information.

Data

Policy information about availability of data

All manuscripts must include a data availability statement. This statement should provide the following information, where applicable:

- Accession codes, unique identifiers, or web links for publicly available datasets
- A description of any restrictions on data availability
- For clinical datasets or third party data, please ensure that the statement adheres to our policy

Reference genome assembly and annotation files of TM-1 (v3.1), UGA230 (v1.1), UA48 (v1.1) and CSX8308 (v1.1) genomes are available at https://phytozome-next.jgi.doe.gov/. All raw sequence reads have been deposited in the NCBI SRA database under BioProject accessions PRJNA1071074, PRJNA1071075, PRJNA1071076 and PRJNA1071077.

Research involving human participants, their data, or biological material

Policy information about studies with human participants or human data. See also policy information about sex, gender (identity/presentation), and sexual orientation and race, ethnicity and racism.

Reporting on sex and gender	N/A
Reporting on race, ethnicity, or other socially relevant groupings	N/A
Population characteristics	N/A
Recruitment	N/A
Ethics oversight	N/<br>N/A

Note that full information on the approval of the study protocol must also be provided in the manuscript.

Field-specific reporting

Please select the one below that is the best fit for your research. If you are not sure, read the appropriate sections before making your selection.

☒ Life sciences ☐ Behavioural & social sciences ☐ Ecological, evolutionary & environmental sciences

For a reference copy of the document with all sections, see nature.com/documents/nr-reporting-summary-flat.pdf

Life sciences study design

All studies must disclose on these points even when the disclosure is negative.

Sample size	Sample size per group or condition was determined based on the minimum number of biological replicates (n=3) required to perform differential expression analysis as per DESeq2 R package (Love et al., 2014) and previously published literature.
Data exclusions	Samples were excluded if they failed at the library preparation stage or those that displayed poor correlation (Pearson correlation R < 0.85) between biological replicates.<br>Only SNPs with ≤20% missing data and minor allele frequencies >0.005 were retained, rest excluded.
Replication	For RNA-seq, data from 3 independent biological samples per condition was collected. We used 4 individual plants per cultivar for fiber quality and yield experiment. For fiber histology and microscopy experiment, a minimum of 60 samples per cultivar were used.
Randomization	Order of sample processing for library preparation and sequencing were processed in multiple batches as and when they were received from collaborating laboratories, kind of randomization in itself, but following stringent standardized protocols.
Blinding	No blinding took place. To alleviate any complications from non-blinded analyses all samples were analyzed simultaneously in the same manner regardless of their condition/origin.

Reporting for specific materials, systems and methods

We require information from authors about some types of materials, experimental systems and methods used in many studies. Here, indicate whether each material, system or method listed is relevant to your study. If you are not sure if a list item applies to your research, read the appropriate section before selecting a response.

Materials & experimental systems

n/a	Involved in the study
☒	☐ Antibodies
☒	☐ Eukaryotic cell lines
☒	☐ Palaeontology and archaeology
☒	☐ Animals and other organisms
☒	☐ Clinical data
☒	☐ Dual use research of concern
☐	☒ Plants

Methods

n/a	Involved in the study
☒	☐ ChIP-seq
☒	☐ Flow cytometry
☒	☐ MRI-based neuroimaging

Dual use research of concern

Policy information about dual use research of concern

Hazards

Could the accidental, deliberate or reckless misuse of agents or technologies generated in the work, or the application of information presented in the manuscript, pose a threat to:

No	Yes
☒	☐ Public health
☒	☐ National security
☒	☐ Crops and/or livestock
☒	☐ Ecosystems
☒	☐ Any other significant area

Experiments of concern

Does the work involve any of these experiments of concern:

No	Yes
☒	☐ Demonstrate how to render a vaccine ineffective
☒	☐ Confer resistance to therapeutically useful antibiotics or antiviral agents
☒	☐ Enhance the virulence of a pathogen or render a nonpathogen virulent
☒	☐ Increase transmissibility of a pathogen
☒	☐ Alter the host range of a pathogen
☒	☐ Enable evasion of diagnostic/detection modalities
☒	☐ Enable the weaponization of a biological agent or toxin
☒	☐ Any other potentially harmful combination of experiments and agents

Plants

Seed stocks

Gossypium hirsutum L. acc. TM-1 (1008001.06), UGA230, UA48 and CSX8308 were grown in a greenhouse at Clemson University. UGA230 (PVP 201500309 or UGA 2004230) is a conventional upland cotton cultivar that was developed and released by the University of Georgia Agricultural Experiment Station in 2009.
UA48 (Reg. No. CV-129, PI 660508) is a conventional upland cotton cultivar that was released by the Arkansas Agricultural Experiment Station in November 2010.
CSX8308 (Siokra 250) was developed in a planned breeding program at CSIRO Australia by crossing two proprietary breeding lines 64005-56OL x 64014-338NL.

Novel plant genotypes

Describe the methods by which all novel plant genotypes were produced. This includes those generated by transgenic approaches, gene editing, chemical/radiation-based mutagenesis and hybridization. For transgenic lines, describe the transformation method, the number of independent lines analyzed and the generation upon which experiments were performed. For gene-edited lines, describe the editor used, the endogenous sequence targeted for editing, the targeting guide RNA sequence (if applicable) and how the editor was applied.

Authentication

Describe any authentication procedures for each seed stock used or novel genotype generated. Describe any experiments used to assess the effect of a mutation and, where applicable, how potential secondary effects (e.g. second site T-DNA insertions, mosiacism, off-target gene editing) were examined.
